# Randomized control trial of computer-based training targeting alertness in older adults: the ALERT trial protocol

**DOI:** 10.1186/s40359-018-0233-4

**Published:** 2018-05-03

**Authors:** Thomas VanVleet, Michelle Voss, Sawsan Dabit, Alex Mitko, Joseph DeGutis

**Affiliations:** 1 Posit Science Corporation, San Francisco, CA USA; 2 Department of Veteran Affairs, VA Medical Center, Martinez, CA USA; 3 Health, Brain and Cognition Lab, University of Iowa, Iowa City, IA USA; 4 Department of Psychological and Brain Sciences, University of Iowa, Iowa City, IA USA; 5 Boston Attention and Learning Laboratory, VA Boston Healthcare System, Boston, MA USA; 6 Harvard Medical School, Department of Psychiatry, Boston, MA USA

**Keywords:** Healthy aging, Computer-based cognitive training, Attention, TAPAT, Plasticity

## Abstract

**Background:**

Healthy aging is associated with a decline in multiple functional domains including perception, attention, short and long-term memory, reasoning, decision-making, as well as cognitive and motor control functions; all of which are significantly modulated by an individual’s level of alertness. The control of alertness also significantly declines with age and contributes to increased lapses of attention in everyday life, ranging from minor memory slips to a lack of vigilance and increased risk of falls or motor-vehicle accidents. Several experimental behavioral therapies designed to remediate age-related cognitive decline have been developed, but differ widely in content, method and dose. Preliminary studies demonstrate that Tonic and Phasic Alertness Training (TAPAT) can improve executive functions in older adults and may be a useful adjunct treatment to enhance benefits gained in other clinically validated treatments. The purpose of the current trial (referred to as the Attention training for Learning Enhancement and Resilience Trial or ALERT) is to compare TAPAT to an active control training condition, include a larger sample of patients, and assess both cognitive and functional outcomes.

**Methods/design:**

We will employ a multi-site, longitudinal, blinded randomized controlled trial (RCT) design with a target sample of 120 patients with age-related cognitive decline. Patients will be asked to complete 36 training sessions remotely (30 min/day, 5 days a week, over 3 months) of either the experimental TAPAT training program or an active control computer games condition. Patients will be assessed on a battery of cognitive and functional outcomes at four time points, including: a) immediately before training, b) halfway through training, c) within forty-eight hours post completion of total training, and d) after a three-month no-contact period post completion of total training, to assess the longevity of potential training effects.

**Discussion:**

The strengths of this protocol are that it tests an innovative, in-home administered treatment that targets a fundamental deficit in adults with age-related cognitive decline; employs highly sensitive computer-based assessments of cognition as well as functional abilities, and incorporates a large sample size in an RCT design.

**Trial registration:**

ClinicalTrials.gov identifier: NCT02416401.

## Background

Healthy aging is associated with a decline in multiple functional domains including perception, attention, short and long-term memory, reasoning, decision-making, as well as cognitive and motor control functions [1–3]. Studies show that cognitive decline is paralleled by the atrophy of the neuromodulatory control machinery that crucially supports synaptic plasticity, and its deterioration is a primary contributor to widespread structural and functional brain changes (e.g., reduction in cortical thickness, alterations in myelination, reduction in blood flow/perfusion, reduced resting state connectivity [4]).

Research in age-related cognitive decline has identified four inter-related classes of deficits that can all be expected to be altered as a consequence of neuromodulatory system atrophy: 1) processing speed loss, marked by the slowing of information processing at every neurological level [5]; 2) loss of inhibitory control, which contributes to impairments in background (noise and distractor) suppression [6–8], 3) degraded perceptual processing [9, 10], which necessarily limits all aspects of higher-order cognitive processing; and 4) deterioration of explicit cognitive and movement control processes [6]. Natural or plasticity-based changes in processing speed arise as a product of perceptual degradation and an increase in neural process noise.

Considered from another perspective, processing speed, distractor suppression and perceptual acuity are all significantly modulated by an individual’s level of cognitive alertness [11]—the control of which also significantly declines with age. Decline in alertness, a function important for sustained attention contributes to increased lapses of attention in everyday life, ranging from minor memory slips to a lack of vigilance and increased risk of falls or motor-vehicle accidents. In fact, a recent study has compellingly shown that sustained attention performance can provide an objective cognitive marker for frailty progression in older adults [12]. Alertness and sustained attention rely on a broad network of regions that include the locus coeruleus (LC) in the brainstem, the right inferior frontoparietal regions, medial prefrontal cortex, and the DMN [13, 14]. The LC synthesizes norepinephrine (NE), an excitatory neurotransmitter intimately involved in arousal, and innervates the entire cerebral cortex as well as subcortical regions. Predominantly right inferior frontoparietal, amygdala, and medial prefrontal regions send feedback projections to the LC, regulating its output. Considering the complexity of this system, age-related decline in alertness and sustained attention may be caused by both reduction in NE neurons in the LC, and/or impaired interactions between the LC and associated cortical regions [15, 16].

Sustained attention, particularly as it relates to the examination of novel stimuli, also depends on the integrity of another key neuromodulator, the nucleus basalis (NB) in the basal forebrain [17, 18]. NB activity is associated with the release of acetylcholine (Ach), and has been shown to play an important role in both the attention-based modulation of cortical activity [18] and the suppression of background noise and distractors [19]. The NB or forebrain/cholinergic/system also suffers age related declines in efficiency as a prelude to neurodegenerative disease and has been shown to be a critical enabler of experience-dependent plasticity [20].

Many additional factors contribute to cognitive decline in healthy aging [21, 22], and prior training studies have targeted – and driven improvements in – age-related deficits (e.g., perceptual processing, speed of processing, working memory and executive function) [23]. However, failure to address the deterioration of processes related to the regulation of alertness and sustained attention (e.g., the modulation of forebrain activities controlled by selective or phasic attention; suppression of background distractors; engagement of attention and reward-system responses that directly regulate synaptic change) can be expected to attenuate or frustrate efforts to improve age-related cognitive decline, and leave the path open for continuing decline [24].

Several experimental behavioral therapies designed to remediate age-related cognitive decline have been developed over the last 10–12 years. However, methodological weaknesses, such as small sample sizes, lack of adequate controls, and limited outcome measures have made it difficult to interpret the meanings of both significant and nonsignificant findings [25–27]. To date, experimental interventions for age-related cognitive decline have differed widely in content, method and dose, and there is an ongoing debate regarding the question of whether training can improve untrained cognitive abilities (i.e., effects that generalize across cognitive domains). Not surprisingly, no treatment has been widely adopted and there is no consensus about which therapy is most effective [28].

Thus, to address current knowledge gaps and target age-related cognitive deficits, we have developed a treatment targeting both sustained (tonic) and moment-to-moment (phasic) aspects of attention (tonic and phasic alertness training, TAPAT). TAPAT is a continuous performance task in which all stimuli are presented at central fixation with several key elements that help older adults to stay engaged such as jittered inter-trial intervals [29, 30], rich, colorful, and novel stimuli, and a response inhibition component. In a preliminary trial of TAPAT [31], healthy older adults were randomly assigned to treatment or an active control group (AC); participants in the treatment group engaged in the computerized, continuous performance training task that required that they remain alert and engaged (tonic alertness), responding quickly to all non-target objects, scenes or sounds while waiting for an unpredictable and infrequent target object or scene [32–34]. Critically, participants were challenged to inhibit the prepotent motor response (phasic alertness) when they saw the target stimulus. Following ~ 5 h of training over six weeks, participants demonstrated improvements in inhibitory control (withholding response) and sustained attention (reduction in response time variability) in the training task. Also, participants in the TAPAT training group showed significant improvement in speeded visual selective attention compared to participants in the AC group. Moreover, the results show that training related improvement in sustained attention and inhibitory control generalize to untrained, challenging measures of executive function (e.g., working memory, fluency, set-shifting). Magnitude of improvement in the training task (target accuracy) was significantly correlated with the magnitude of improvement in executive function. Finally, when participants were trained in a secondary perceptual learning task, learning rates were accelerated and the level of improvement achieved were greater when that training was preceded by a short epoch of TAPAT training, compared to the AC. The results are consistent with recent, parallel studies in which participants with acquired brain injury also showed improvements in spatial and non-spatial attention, and executive functions [35], following TAPAT training versus participants in wait-list or active control training conditions [32–34].

### Aims and hypotheses

The aim of the current study is to test the effectiveness of a longer (12–16 weeks) version of computer-based TAPAT training to improve cognitive abilities (e.g., attention, working memory, executive function), functional status and quality of life of individuals with age-related cognitive decline as compared to a computer-based active control. Secondary aims include measuring the degree to which these effects persist after a three-month no-contact period, and also demonstrate equivalency in safety reported between the treatment and control groups (FDA requirement).

## Methods

### Overall design and timeline

**Fig. 1:**
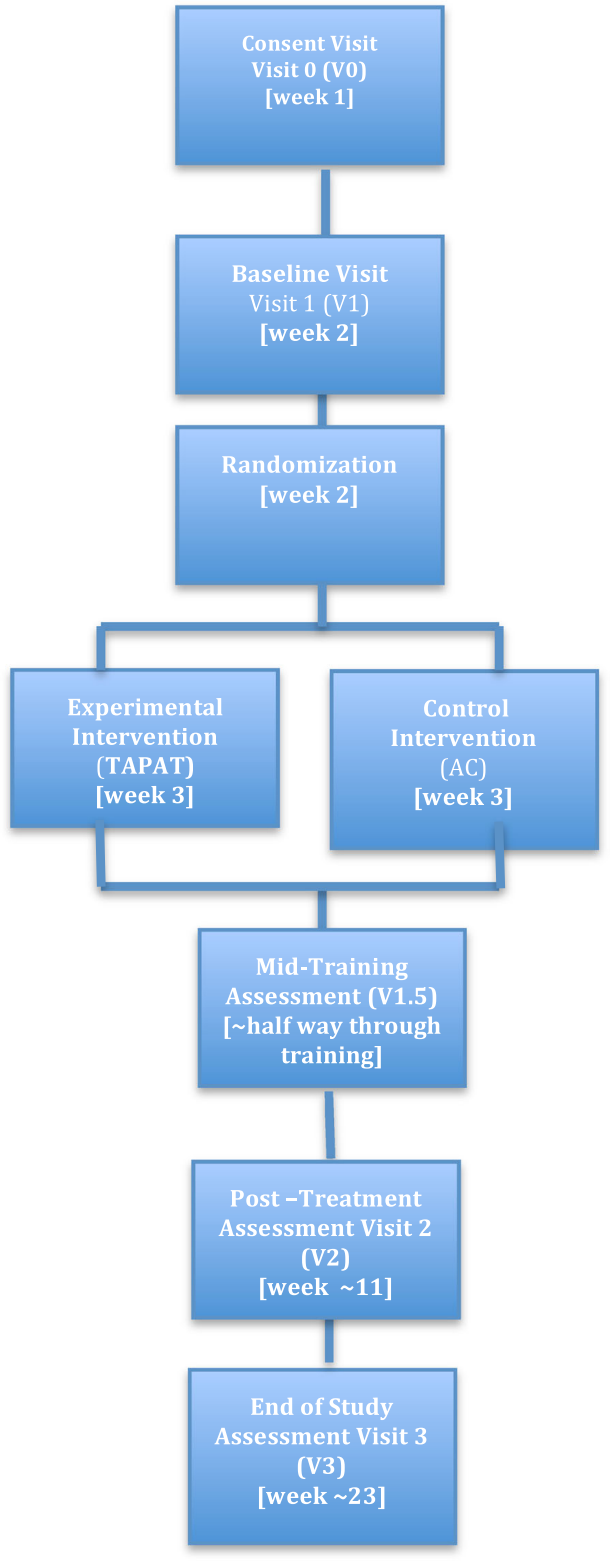
Study Outline

The current study will employ a multi-site, longitudinal, blinded randomized controlled trial (RCT) design with a target sample of 120 older adults without dementia or MCI (see inclusion criteria below). Sixty older adults completing TAPAT will be compared to sixty older adults completing the active control condition (computer-based games; see Fig. 1). Total participation time is approximately 6 months and includes 5 in-person assessment sessions. Assessments will be performed at University of Iowa (Iowa City, IA) and VA Boston Healthcare System (Boston, MA). The first assessment session (V0) involves screening for eligibility (see inclusion/exclusion below). If the participant is eligible, baseline assessments are administered in a second visit (V1) to characterize their cognitive and functional abilities before training. After the baseline assessment, older adults are randomized to either the TAPAT or control training program and complete approximately 12–16 weeks of in-home training, while monitored and coached by a research assistant (*cognitive remediation coach*). Participants assigned to the experimental software treatment program will be randomly assigned to receive performance feedback (version A.1 vs. A.2) in the first or the second half of training. Participants will then be asked to complete a mid-training assessment (V1.5) and will be administered a subset of assessments comprised of the secondary outcome: mindfulness. At the mid-training assessment, participants who were randomized to the experimental program will switch program versions (i.e., participants that trained on version A.1 will begin version A.2, and vice versa), such that participants train on both versions. To measure potential training-related improvements, participants will be assessed immediately after the completion of the total training (V2) by an assessor that is blind to group affiliation. To measure the persistence of potential training-related improvements, participants are also assessed after a three-month no-contact period (V3), again by an assessor blind to group affiliation. After this visit, participant activities are completed and trial participation ends.

### Study population

The study population is comprised of healthy older adults with axiomatic age-related cognitive decline. Due to the average age of individuals with age-related cognitive decline (i.e., 60–90 years old), we expect this population to have additional challenges, including but not limited to vision and/or hearing difficulties, motor difficulties, and other unrelated, pre-morbid medical complications. We will only enroll individuals for whom these complications will not interfere with assessment procedures or completion of the training programs.

The following inclusion/exclusion criteria will be determined through our screening procedures during V0, which includes structured interviews, as well as computerized and standardized neuropsychological assessments of attention, cognition and functional abilities.

#### Inclusion criteria

1. 65 years of age or older at the time of consent
2. Fluent spoken English by the age of 12 in the judgment of the consenting clinician or as verified via participant interview.
3. Participants must have adequate sensorimotor capacity to participate in the trial, including visual capacity, auditory capacity, and motor capacity adequate to control a computer mouse.
4. Age-related cognitive status will be confirmed through performance on the Montreal Cognitive Assessment (MoCA). Participants must score a 26 or above on the MoCA.

#### Exclusion criteria

1. Diagnosis of PTSD, depression or other psychologically diagnosable emotional disorder as verified via participant self-report, and performance on the Geriatric Depression Scale (GDS ≥ 20). If the participant has previously had a psychologically diagnosable Axis I emotional disorder and they have not experienced an episode for more than 2 years, they may be included.
2. Diagnosis of an illness or condition with known cognitive consequences (e.g., schizophrenia, bipolar disorder, cancer, multiple sclerosis) will be excluded due to the confound with cognitive impairment from those conditions. If the participant has received 5 or less doses of chemotherapy in the two years preceding potential enrollment, they may be included. These exceptions are up to the discretion of the Study Principal Investigator and Site Principal Investigators.
3. Participants who have answered ‘yes’ to:

- Question 5 (Active Suicidal Ideation with Specific Plan and Intent) on the Columbia-Suicide Severity Rating Scale (C-SSRS) or,
- ‘Yes’ to any of the suicide-related behaviors (actual attempt, interrupted attempt, aborted attempt, preparatory act or behavior) on the “Suicidal Behavior” portion will be excluded from the study *if the ideation or behavior occurred within two months from Participant’s date of consent* (as recommended by the FDA for treatment trials.) Participants excluded for this reason will be referred for appropriate treatment. Further, participants meeting this criteria at any time throughout the study will be asked to complete a final assessment, if appropriate, then withdrawn from the study and referred for appropriate treatment.

4) Current or significant past history of substance abuse in the judgment of the Site PI.
5) Difficulty completing assessments and/or comprehending requirements of the trial (e.g., following verbal instructions).
6) Enrollment in a concurrent clinical trial involving an investigational pharmaceutical, nutraceutical, medical device or behavioral treatment that could affect the outcome of this study will be excluded. Participants will not be excluded for participation in conventional treatments (e.g., physical or occupational therapy) or use of prescribed medications.

#### Recruitment

Each study site has pre-existing recruitment methods specific to the normally aging population and all sites screen hundreds of potential participants per year. Further, Site PIs Dr. Joe DeGutis (Harvard Medical School) and Dr. Michelle Voss (University of Iowa), are internationally recognized experts in geriatric neuropsychology/psychiatry and have access to large, established cohorts of older participants. Sites will also make specific efforts to reach out to community dwelling individuals with age related cognitive decline who are not regular visitors to their clinics in order to improve the prospective validity of the participant sample. Additional recruitment methods include public presentations about the trial, brochures and flyers that describe the study and include information regarding inclusion/exclusion criteria as well as mechanism for indicating interest and communicating desire to be contacted. All materials used for advertising or recruitment will have received IRB approval prior to implementation, and the study sites will conform to recruiting standards established at each site.

### Repeated assessment battery

Once a participant is deemed eligible for participation based on their V0 results, they are next scheduled for their baseline session on the repeated assessment battery (V1), which takes approximately two hours. After completing this baseline assessment, participants are randomly assigned to either experimental or control training conditions (see below). Then, following the program set-up visit and after completing the first half of the training sessions, they will be administered an abbreviated mid-training assessment (V1.5) comprised of mindfulness-related measures. Immediately after total training is completed, participants are re-administered the baseline assessment battery (V2) and again after a three-month no contact period (V3).

### Primary and secondary outcome measures

#### Primary outcome

The primary outcome is a composite executive function measure composed of the neuropsychological assessments shown to capture executive functions that were not the focus of training (i.e., assessments that do not directly require sustained inhibitory control, the mode of training), including:

- Trails B (time to complete, raw score)
- DKEFS verbal fluency (total accurate switches, raw score)
- Auditory Consonant Trigrams (sum of accurate recalls following 9 s, 18 s and 32 s delays, raw scores)
- WAIS Digit Span (sum of accurate backward recalls, raw score)
- Attentional Blink task (second target accuracy, raw score)
- Category Switch task (reaction time: incongruent – congruent trials, raw scores)

The composite will be calculated by z-transforming each score and averaging across the six measures.

The primary composite executive function outcome is comprised of executive function measures. First, the Attentional Blink Task, in which participants must identify two target characters presented in rapid succession amongst related distractors. This task assesses participants’ attentional resource allocation distributed over time, as well as working memory encoding and distractor inhibition. We will also include Trails B, a common set-switching task in which participants must rapidly alternate between sequencing letters versus numbers. The Auditory Consonant Trigrams (ACT) test, a complex working memory task requiring participants to remember three letters over delay periods of 9, 18, or 36 s while counting backwards by threes from a given number. Also, the WAIS-IV Digit Span task backwards recall subtest, will be used to assess complex working memory span; participants are required to recall an increasing span of numbers in reverse order to that presented. Finally, participants will be required to complete a speeded Category Switch task, in which explicit rules (e.g., color, shape) direct the participant to accurately and rapidly identify and categorize serially presented objects.

#### Secondary outcomes

The first set of secondary outcomes comprises measures of cognition, learning and memory; primarily standardized neuropsychological assessments, including:

- Gradual onset Continuous Performance Task (target accuracy, raw score)
- Stop Signal task (time interval for successful stop, raw score)

- Flanker task (reaction time: incongruent flanker trials – congruent flanker trials, raw scores)
- Cross-modal Stroop – Mixed Signals (reaction time: response conflict trials, raw score)
- WAIS IV Digit Span, forwards and sequencing (raw scores)
- Spatial Working Memory task (accuracy, raw score)
- DKEFS Verbal Fluency, phonemic and semantic subtest (raw scores)
- Reinforcement Learning task (bias index)
- WMS IV Logical Memory I & II immediate recall (sum of learning trials, raw score)
- WMS IV Logical Memory I & II delayed recall (sum of recall trials, raw score)

Even though some of these measures may engage executive functions (e.g., gradual onset continuous performance task may engage inhibitory control), we chose not to include measures that were very similar to the training in the primary outcome. This is an effort to avoid the criticism that the primary outcome measure is overly influenced by improvements in near transfer tasks.

The second set of secondary outcomes comprise measures of functional performance, quality of sleep, quality of life and mindfulness; and include self-report and directly-observed functional performance measures:

- Walking Behavior Measure (raw score)
- Self-efficacy Assessment (two scales, raw scores)
- Falls Efficacy scale (raw score)
- Timed Up and Go task –TUG (time to completion, raw score)
- SF-12 (raw score)
- Cognitive Failures Questionnaire (raw score)
- Pittsburg Sleep Quality Index (raw score)
- Mindful Attention Awareness Scale – MAAS (raw score)
- Breath Counting task (accuracy, raw score)

**The secondary outcome –** cognition is comprised of measures of cognition including response control, captured by the Gradual Continuous Performance Task (GradCPT) [36, 37]; participants must sustain engagement (i.e., no inter-trial break), frequently respond to foil images and exert inhibitory control to overcome the proponent motor response when presented with an infrequent target image (i.e., withhold response). Also, the stop signal task, in which participants must inhibit a triggered response (i.e., go signal) when a *stop signal* (e.g., tone or color) is presented after the onset of the trigger. The Flanker task [38], in which participants must quickly identify the direction of a central arrow flanked by either congruent (i.e., facing the same direction as the central arrow) or incongruent arrows; and necessarily inhibit the influence of incongruent flanking stimuli. A cross-modal Stroop task, Mixed Signals (BrainHQ.com), requires participants to rapidly overcome interference from competing responses and information from conflicting stimulus modalities. WAIS-IV Digit Span (forward and sequencing subtests) is a verbal working memory span task, where the subject is given a list of numbers to remember and instructed to repeat the numbers either in the same order (digits forward subtest) or canonical order (sequencing subtest). In the Spatial Working Memory task participants are presented with several possible target locations displayed centrally along the vertical meridian. Following each trail, the participant is presented with a probe to a single location to which they must decide if the given location was included in the possible target locations (yes/no) [39]. The DKEFS Verbal Fluency, semantic and phonemic cueing subtests will also be included to examine broad executive function ability requiring spontaneous and rapid generative fluency. Scoring is based on the number of accurate replies per cue type (phonemic, semantic) and response characteristics (e.g., intrusions, perseverative errors) are also scored.

**The secondary outcome -** learning and memory is comprised of two tasks that examine learning rate via the Reinforcement Learning Task, designed and developed by Dr. Erin Rich [40], as well as acquisition and short and long delay free, cued and recognition memory performance via Logical Memory I & II (WMS-IV).

**The secondary outcome** - functional performance is comprised of performance on a directly observed measure as well as self-report questionnaires. For self-report, we will use the Walking Behavior measure, in which participants are asked to report the numbers of times (and duration of time) that they spent brisk walking per week. The Self-efficacy assessment, which is comprised of two measures, way-finding self-efficacy and self-efficacy for walking [41], will provide insight into participants’ perceived sense of direction [42] and self-efficacy for navigation [43]. The Falls Efficacy Scale [44] provides a valid and reliable measure of *confidence* when engaging in various daily activities (e.g., get out of bed, take shower). Finally, we will employ a directly observed measure, the Timed Up and Go (TUG) task [45], which assesses general mobility and balance.

**The secondary outcome -** quality of life is comprised of performance on the Short-Form 12 (SF-12v2) [46], a measure of Health-Related Quality of Life. This assessment serves as a measure of the impact of program use on the participants’ own view of their impairment and function (i.e., quality of life). The Cognitive Failures questionnaire will also be administered to evaluate common errors in daily activities that may affect quality of life.

**The secondary outcome -** quality of sleep is comprised of performance on the Pittsburg Sleep Quality Index. This measure has been used in many outcomes studies including a prior study of TAPAT outcomes in veterans with PTSD in which it effectively captured improvements in sleep quality post-training compared to a waitlist control group [47].

**The secondary outcome –** mindfulness includes two measures that will be included in the mid-training assessment to assess mindfulness and awareness. The Mindful Attention Awareness Scale (MAAS) is a valid measure for mindfulness. The second is a breath-counting task [48], serving as a behavioral measure of mindfulness.

### Randomization

Participants will be randomized after the baseline visit and before the planned first day of program use. All V0 and V1 data for each participant must be fully monitored, with all queries resolved, before randomization may take place. We will use a randomization server (sealedenvelope.com) that implements the random permuted blocks within strata procedure [49, 50] with strata as age (*Older Adults 65–74* or *Older Adults* ≥ 75 years of age), allocation ratio is 1:1. Participants who are randomized to the experimental treatment group will also be randomized to either receive version 1 (experimental treatment with performance feedback) or version 2 (experimental treatment without performance feedback); at the mid-training assessment, participants will undergo a reversal of the version type such that all participants completing the entirety of the training will have trained on both versions of the experimental training program. The Site Coordinating Center will issue a randomization assignment at the appropriate time. This approach represents a best practice approach to randomization, implementing an automated centralized group assignment procedure with allocation concealment, and effective separation of sequence generation and allocation concealment.

### Blinding

#### Un-blinded site roles

At each site, cognitive remediation coaches are un-blinded in order to provide support for participants using their assigned programs. They will be distinct from staff administering and scoring assessments. Additionally, site sub-investigators authorized to register participants within the ALERT system will remain un-blinded and may not participate in the assessment, evaluation, or follow-up of study participants.

#### Blinded site roles

All site staff responsible for the administration and scoring of participant assessments will remain blinded to participant treatment. Site Principal Investigators will be required to complete a Delegation of Authority Form prior to the start of the study, indicating which activities individual site research team members will be authorized to complete. Site Principal Investigators will also remain blinded.

To prevent un-blinding, the following controls will occur at the site level:

1. The treatment condition and the control condition will be identified as “Treatment A” and “Treatment B”;
2. Participants will be reminded not to discuss details related to treatment with psychometricians and/or clinical evaluators during the informed consent process as well as prior to initiation, and at the conclusion of, each assessment visit;
3. Site personnel will be instructed to not discuss details of either treatment arm during open participant groups or forums;
4. Sites will be required execute the protocol in a manner that minimizes the possibility of accidental un-blinding of psychometricians or clinical evaluators (e.g. unintended viewing of treatment sessions);
5. Sites will be asked to post signage in appropriate areas throughout the facility reminding staff and participants to not discuss treatment details in open locations.

At the half-way point of the trial, and at the end of the trial, psychometricians will be asked questions designed to evaluate the integrity of the blinding procedures employed throughout the study.

### Description of treatment program

*The Experimental Treatment Program* is a computerized cognitive remediation program consisting of a set of specific cognitive exercises. Participants perform tens to hundreds of trials over the course of a session, with auditory and visual feedback and rewards to indicate if the trial was performed correctly or incorrectly (version A.1). After each session, the difficulty of the next session is updated (e.g., less inter-stimulus-interval jitter) to ensure that each participant is appropriately challenged. Summary screens including game metrics (points, levels) and exercise metrics (usage, progress) are shown to the participant at the end of each session (version A.1).

**Fig. 2:**
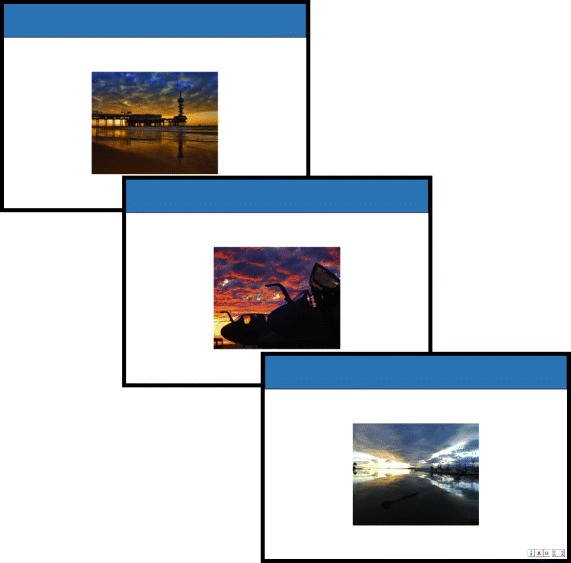
A visual, within-category version A.2 of TAPAT (without feedback)

There are two versions of the experimental software treatment program, TAPAT. One version provides participants with real-time feedback on their training performance (version A.1), while the other version does not provide participants with performance feedback (version A.2). A description of exercises in experimental treatment program is as follows:

The primary component of the experimental treatment program, *Tonic and Phasic Alertness Training (TAPAT),* is comprised of three variations designed to improve the individual’s intrinsic regulation of alertness and executive control. Specifically, the ability to sustain attention and respond to successively presented stimuli in a consistent manner (i.e., low reaction time variability), and ability to inhibit the proponent motor response when a target is presented. Exercise variants: 1) *Freeze Frame*, a visual category-nonspecific version; 2) a visual, within-category version; 3) a visually engaging, category-nonspecific version (Fig. 2).

**Fig. 3:**
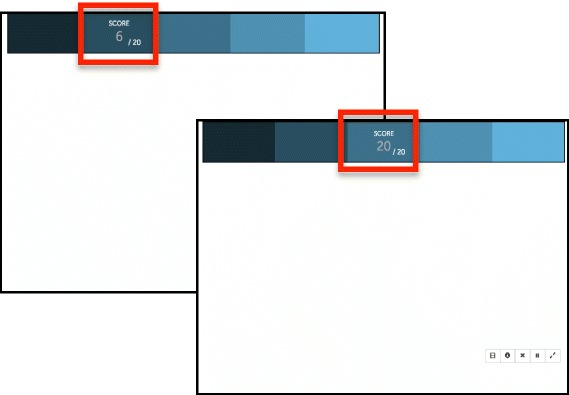
Version A.1 of TAPAT with performance feedback provide every ~ 2 min in the form of an accumulated score (max = 20 per two minutes; total score max = 100)

In version A.1, real-time performance feedback is provided in each exercise at five distinct times, at 20% completion intervals for that exercise. Participants are instructed to be mindful of their performance and to make an effort to maintain a performance goal of 20/20 for each interval (Fig. 3).

All training sessions will consist of the three variations of TAPAT to provide variety during each training session. A summary of the training schedule is provided in Table 1.

**Table 1:** Training Schedule for a participant randomized to receive TAPAT with feedback in the first half of training

Sessions 1–18	TAPAT with Feedback (v.A.1)	(32 min/session)
	TAPAT (10 min) > 60 s break> TAPAT (10 min) > 60 s break > TAPAT (10 min)	
V1.5	Mid-training assessment (V1.5) Subset of assessments administered in-person.	
Sessions 19–36	TAPAT without Feedback (v.A.2)	(32 min/session)
	TAPAT (10 min) > 60 s break> TAPAT (10 min) > 60 s break > TAPAT (10 min)	

*Active Control Program (commercially-available computer games):* The active control program is composed of 6 commercially available computer games and is designed to: 1) be a face-valid approach to treating cognitive remediation age-related decline (analogous to crossword puzzles for age-related cognitive decline), ensure that participants remain blind to group affiliation, and match the experimental treatment program in halo or expectation-based influence on performance in neuropsychological outcome measures; 2) match the experimental treatment program in overall program use intensity, time-spent attending, delivered rewards, and overall engagement; and 3) provide a comparison group that matches the experimental treatment group on the aforementioned attributes, but without the known therapeutic elements.

### Foreseeable risks, Risk Management & Emergency Response

Participation in the study presents minimal risk. The probability and magnitude of harm or discomfort anticipated in this research study are not greater than those ordinarily encountered in daily life or during the performance of routine physical or psychological examinations or tests. Study participation may be discontinued when a participant voluntarily withdraws from the study (e.g. due to scheduling issues or changing residence) or when they express discomfort with any of the study procedures (e.g. excessive frustration, boredom, or eye strain). To improve adherence to the interventions, weekly check-ins were performed to monitor participants’ progress, motivate and provide feedback, and allow participants to voice any adverse effects.

Serious adverse effects from prior studies of the treatments under study have not been reported. The protocol details potential risks related to study participation and includes assessments of depression and suicidality at baseline, week 13, and week 26 (end-of-study). Participants will be encouraged to report any adverse effects occurring during the duration of the study to the point of contact within research staff. PSC does not provide compensation for research-related injuries and will not reimburse or pay medical expenses for the treatment of research-related injuries.

We will conservatively follow guidelines for medical devices in the reporting of adverse events in this trial, which defines unanticipated adverse device effects (UADEs) in The Code of Federal Regulations in 21 CFR 812.3(s) as any serious adverse effect on health or safety of any life-threatening problem or death caused by, or associated with, a device, if that effect, problem, or death, was not previously defined in nature, severity or degree of incidence in the investigational plan or application (or supplementary plan or application). Furthermore, an effect is classified as an UADE if it is judged by the Site PI to be a serious problem associated with a device that related to the rights, safety, or welfare of participants. We will operationalize this definition of a serious problem as one that results in any of the following outcomes: death, life-threatening situation, inpatient hospitalization atypical of the participant’s diseases condition, persistent or significant disability/incapacity; or any other adverse event that, based upon appropriate medical judgment, may jeopardize the participant’s health or the health and well-being of all participants enrolled in the study.

Site Study Coordinators, Psychometricians, and Cognitive Remediation Coaches will ask about any UADEs during each contact with participants and will be alert to any volunteered UADEs. All UADEs will be documented on a standardized eCRF and will be classified by the blinded Site PI to their degree of seriousness and their relationship to the study software.

All UADEs, whether or not we believe them to be related to the protocol, will be reported to the Site IRB according to the investigational site’s IRB procedures and to Study PI (Thomas VanVleet) as soon as possible, but in all cases within 10 working days of the event. PSC will circulate UADE reports to the Research Monitor and all trial sites within 10 working days of receipt. Each study site will report immediately to: 1) University of Iowa IRB, 2) PSC IRB, or 3) At the Boston VA, UADEs will be reported to the VA Central IRB electronically through a secure SharePoint system and confirmed by phone to 202–461-1859.

### Data management

We will take all standard and appropriate steps to protect the privacy and confidentiality of participants in this trial. All data collected in this study (with one small exception for electronic usage data captured directly from program use, discussed below) will be from clinician- or psychometrician-administered structured interviews, neuropsychological or functional assessments. Following consent, each participant will be assigned a standardized Participant Identification Number (PIDN). All study-related data will be recorded into a secure, web-based electronic case report form (eCRF) at each site. This system meets all relevant privacy and security standards for electronic clinical trial data entry and storage, as well as the Health Insurance Portability and Accountability Act (HIPAA) standards for confidentiality and privacy. All eCRF data entry will use the only PIDN and not the participant name, Each site will transcribe and upload de-identified data and de-identified source documentation into the study database for the purpose of data monitoring.

To help meet the highest standard of clinical trial management, a Data Safety Monitoring Board (DSMB) independent of the Sponsor and/or study Principal Investigator(s) was established to provide independent overview of safety data, aggregate study data, data management processes, and a priori analyses of primary and secondary endpoints. A DSMB Chairperson shall be appointed in advance to document member attendance and study outcomes (including the statistical methods employed to complete analyses).

### Data analysis

The data analysis plan a priori defines a primary intent-to-treat (ITT) population, a set of secondary evaluation populations, a primary outcome measure, a set of secondary outcome measures, a single primary evaluation time point, a secondary evaluation time point, a primary statistical analysis methodology, a criterion for statistical significance, and guidance for interpretation of results. There will be no interim analyses.

The primary ITT population is defined as all participants who complete a set-up (V1) visit. Note that this includes all randomized participants except those who drop/withdraw post-randomization and pre-setup visit. In total, there are three a priori defined analysis populations, including a primary analysis population (i), a secondary analysis populations designed to compare effect sizes in populations with no missing data (ii) and a tertiary analysis population who completed all training visits (iii)

i. *Intent to Treat (ITT) population:* This is the a priori primary analysis population, defined as including all randomized participants who completed a V1.
ii. *Intent to Treat (ITT) Fully-Evaluable (FE) population:* This is a secondary analysis population, defined as including all members of the ITT population that complete a V2 visit (the ITT-FEV2 population), or all members of the ITT population that completed a V3 visit (the ITT-FEV3 population). Note that a participant may complete a specific visit but have missing data for a test in which case the participants is in the overall FE population but does not contribute data to the FE population for that visit, e.g., the number of evaluable cases for a specific test on a specific visit may be smaller than the FE population for that visit because of missing data.
iii. *Intent to Treat (ITT) Fully-Trained (FT) population:* This is a secondary analysis population, defined as including all members of the ITT-FE population who complete a target number of training visits (65 visits). Note that the FT populations are strict subsets of the FE populations; a person who completes the target number of training visits but does not complete the evaluation visit is not a member of the FT population.

The primary executive function outcome measure is a composite measure composed of the main neuropsychological assessments, specifically including

- Trails B (raw score) – Trails A (raw score)
- DKEFS verbal fluency (switching raw score)
- Auditory Consonant Trigrams (sum of 9 s, 18 s and 32 s delay raw scores)
- WAIS Digit Span (backward raw score)
- Attentional Blink task (second target accuracy, raw score)
- Category Switch task (incongruent – congruent trials reaction time)

### The secondary outcome measures are:

- Gradual onset Continuous Performance Task (target accuracy, raw score)
- Stop Signal task (time interval for successful stop, raw score)

- Flanker task (reaction time in incongruent flanker trials – congruent flanker trials, raw scores)
- Cross-modal Stroop – Mixed Signals (reaction time in response conflict trials, raw score)
- WAIS IV Digit Span, forwards and sequencing (raw scores)
- Spatial Working Memory task (accuracy, raw score)
- DKEFS Verbal Fluency, phonemic and semantic subtest (raw scores)
- Reinforcement Learning task (bias index)
- WMS IV Logical Memory I & II immediate recall (sum of learning trials, raw score)
- WMS IV Logical Memory I & II delayed recall (sum of recall trials, raw score)
- Walking Behavior Measure (raw score)
- Self-efficacy Assessment (two scales, raw scores)
- Falls Efficacy scale (raw score)
- Timed Up and Go task –TUG (time to completion, raw score)
- SF-12 (raw score)
- Cognitive Failures Questionnaire (raw score)
- Pittsburg Sleep Quality Index (raw score)
- Mindful Attention Awareness Scale – MAAS (raw score)
- Breath Counting task (accuracy, raw score)

The primary analysis time point is V2, the post-training assessment. The secondary analysis time point is V3, the follow-up assessment.

The primary statistical analysis methodology is a linear mixed model approach. We will first compare treatment and active control groups in the ITT population to determine if any differences in baseline demographic, characterization, outcomes variables, or total program use time remain after the randomization process. Any such factors that show trend level significant differences (*p* < 0.1) will be noted and used as covariates in the linear mixed model analysis. We will examine the data from each outcome measure using a linear mixed model with group and time as fixed factors, site as a random factor, and co-variates as necessary from the baseline analysis. Missing data will be handled with an iterative maximum likelihood procedure to optimally estimate model parameters. The key value for significance will be the group by time interaction factor for the model.

The criterion for statistical significance is *p* < 0.05. Results with *p* < 0.1 will be described as trends.

To evaluate the effect of the intervention on executive function we will conduct the analysis based on the pre-training (baseline) and post-training data. Finding a significance level of *p* < 0.05 on the primary executive function outcome measure will support that the intervention improves executive function in the population.

To evaluate the endurance of effects following completion of the intervention we will conduct the analysis based on the pre-assessment (baseline) and follow-up assessment data. We will conduct confirmatory analyses based on the post-assessment (completion) and follow-up assessment data to evaluate the magnitude of change (further increase or decline) over the no-contact follow-up period.

To evaluate and identify specific populations of treatment responders and non-responders we will conduct exploratory data analyses post-hoc to examine predictors of treatment gain and predictors of lack of treatment gain based on baseline participant demographic, cognitive, and functional measures, as well as on learning rate and plateau performance measures derived over the course of the intervention usage. We will initially employ single regression models to identify variables that predict change in cognitive and functional assessments, and then use generalized linear modeling to explore combinations of predictors of treatment gain or lack of gain.

### Sharing research results

We will share the overall study results with all participants who enrolled in the study when such results are completed and accepted for publication. We will create a lay-person oriented summary of study results to be mailed to each participant at the end of the trial. At enrollment, two pieces of information are collected that, if appropriate, we will share with the participant to ensure they are receiving appropriate health care, include depression status, and suicidal intent. Any participant screening positive for any of these medical issues will be referred to ensure they are receiving appropriate treatment. We do not intend to share individual assessment data with participants, as the assessment battery is not intended to be the type of comprehensive battery a rehabilitation psychologist would use to guide treatment. Any participant interested in such comprehensive assessment will be referred to an appropriate clinician.

## Discussion

Several therapeutic approaches to improve age-related cognitive decline have been developed, including compensatory strategies (e.g., mnemonic memory aids [51], working memory skills [52, 53], speed of processing [54, 55], perceptual learning [9, 56], and multitasking [57] among others [58]. However, few cognitive training approaches have directly targeted alertness in older adults [25] and long term effects are unclear. In the current study, we will examine whether a novel alertness training approach (TAPAT) will benefit attention and executive functions in older adults, as previously shown in acquired brain injury. We hypothesize that the TAPAT group will show greater benefits in these cognitive domains relative to an active control group and that these benefits will transfer to improvements in functional abilities. The ALERT protocol will also help determine whether TAPAT is effective in creating lasting deficit reductions and functional improvements that contribute to an improved standard of care.

### Strengths

TAPAT targets a key neuromodulatory function, alertness, that is fundamental to many lower- and higher-order cognitive operations known to decline in older adults. Further, the vitality of neuromodulatory centers have been shown critical for neuroplasticity. Thus, improving cognitive function and instigating lasting neurological changes is dependent on maintaining a vital level of alertness. Our previous studies have shown that TAPAT is effective in improving spatial and non-spatial attention, as well as executive functions (e.g., set switching) for a wide range of older adults. In addition, TAPAT is simple to perform, easy to remotely monitor (e.g., by a clinician), and is well tolerated by older adults. Because of these qualities, TAPAT is highly scalable (i.e., administration via any Internet connected device), and thus can be readily administered to many older adults with cognitive decline.

The current study is designed to the highest standard of RCT (e.g., blinded and deals with appropriate allocation concealment). Moreover, this protocol will measure immediate as well as lasting benefits of treatment. Lastly, many previous studies lacked statistical power due to a small sample size. ALERT is appropriately powered to detect improvements in targeted cognitive operations. We have statistically powered the study to detect a between groups Cohen’s d effect size of 0.50 on an outcome measure, calculated as the between group difference in the treatment effect means (post-assessment score minus pre-assessment score) divided by the pooled standard deviation of the observed test-retest reliability. This effect size translates, for example, in an improvement of 5.8 points (on an IQ-like index score) within the treatment group versus an improvement of 1.0 point within the active control group, with both groups showing a variance of 10 points (2/3 of a standard deviation, as observed in other composite cognitive performance data) from pre-assessment to post-assessment. Recently published studies that examined the efficacy of the core exercise (TAPAT) in participants with acquired brain injury (*n* = 40) documented effect sizes of 0.75 [32] and 0.69 [33] on the composite attention measure; we believe this is a reasonable estimate of the plausible effect size in this trial. Also, a recent meta-analysis of the effect size of cognitive rehabilitation documented an average between groups effect size of 0.31 [58] in the population of studies used to support the practice guidelines for cognitive rehabilitation [59, 60]. Based on this analysis, we will enroll 120 participants into the trial. We intend to enroll equally from each site (60 per site).

#### Weaknesses

There are also potential limitations. Due to the long duration and multiple components of our study, attrition rates may pose a potential limitation. Furthermore, lack of motivation could prevent timely completions of the self-initiated training program. With a standardized protocol, frequent check-ins, and regular feedback from our research assistants, we aim to limit dropout rate.

## Abbreviations

C-SSRS: Columbia-Suicide Severity Rating Scale
ITT: Intent-To-Treat
SF-12v2: Short Form 12
TAPAT: Tonic and Phasic Alertness Treatment
